# Progress in the Valorization of Fruit and Vegetable Wastes: Active Packaging, Biocomposites, By-Products, and Innovative Technologies Used for Bioactive Compound Extraction

**DOI:** 10.3390/polym13203503

**Published:** 2021-10-12

**Authors:** Mohd Salahuddin Mohd Basri, Nor Nadiah Abdul Karim Shah, Alifdalino Sulaiman, Intan Syafinaz Mohamed Amin Tawakkal, Mohd Zuhair Mohd Nor, Siti Hajar Ariffin, Nur Hamizah Abdul Ghani, Faiqa Shazeaa Mohd Salleh

**Affiliations:** 1 Department of Process and Food Engineering, Faculty of Engineering, University Putra Malaysia, Serdang 43400, Selangor, Malaysia; nadiahkarim@upm.edu.my (N.N.A.K.S.); alifdalino@upm.edu.my (A.S.); intanamin@upm.edu.my (I.S.M.A.T.); zuhair@upm.edu.my (M.Z.M.N.); hajarariffin@upm.edu.my (S.H.A.); nurhamizah@upm.edu.my (N.H.A.G.); faiqazea@upm.edu.my (F.S.M.S.); 2 Laboratory of Halal Science Research, Halal Products Research Institute, Universiti Putra Malaysia, Serdang 43400, Selangor, Malaysia; 3 Laboratory of Biopolymer and Derivatives, Institute of Tropical Forestry and Forest Products (INTROP), Universiti Putra Malaysia, Serdang 43400, Selangor, Malaysia

**Keywords:** fruit waste, vegetable waste, waste valorization, bioactive compound, active packaging, biocomposites, by-product, extraction, thermal processing, non-thermal processing

## Abstract

According to the Food Wastage Footprint and Climate Change Report, about 15% of all fruits and 25% of all vegetables are wasted at the base of the food production chain. The significant losses and wastes in the fresh and processing industries is becoming a serious environmental issue, mainly due to the microbial degradation impacts. There has been a recent surge in research and innovation related to food, packaging, and pharmaceutical applications to address these problems. The underutilized wastes (seed, skin, rind, and pomace) potentially present good sources of valuable bioactive compounds, including functional nutrients, amylopectin, phytochemicals, vitamins, enzymes, dietary fibers, and oils. Fruit and vegetable wastes (FVW) are rich in nutrients and extra nutritional compounds that contribute to the development of animal feed, bioactive ingredients, and ethanol production. In the development of active packaging films, pectin and other biopolymers are commonly used. In addition, the most recent research studies dealing with FVW have enhanced the physical, mechanical, antioxidant, and antimicrobial properties of packaging and biocomposite systems. Innovative technologies that can be used for sensitive bioactive compound extraction and fortification will be crucial in valorizing FVW completely; thus, this article aims to report the progress made in terms of the valorization of FVW and to emphasize the applications of FVW in active packaging and biocomposites, their by-products, and the innovative technologies (both thermal and non-thermal) that can be used for bioactive compounds extraction.

## 1. Introduction

Food waste (FW) is already acknowledged as a major global issue that threatens the long-term viability of the food supply chain [1]. FW in the European Union is estimated to be at 89 million tonnes per year. This is expected to rise by 40% in the next four years [2]. According to the Food and Agriculture Organization of the United Nations [3] and the International Food Policy Research Institute [4] estimated one-third of food produced globally, or 1.3 billion tonnes, is thrown away each year. Over half is generated at the final consumption stage in food services (e.g., restaurants, school canteens) and households [5,6]. The Sustainable Development Goals (SDGs) recognize the importance of this issue. By 2030, SDG Target 12.3 calls for the reduction by half of the per-capita global FW at retail and consumer levels, diminishing food losses in production and supply chains, including post-harvest losses [7]. The call was agreed to by all 193 UN member states, which comes as no surprise.

**Figure 1:**
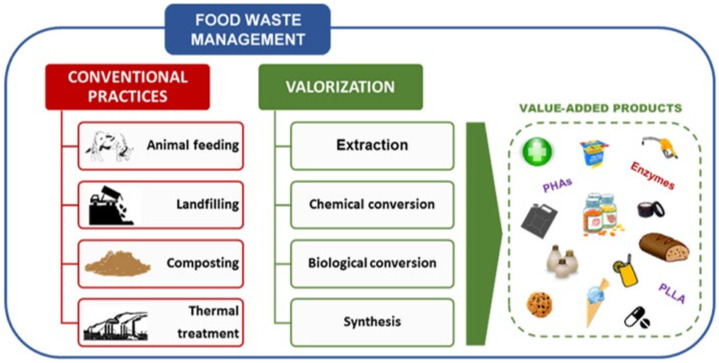
Food waste management practices and the novel or emerging valorization methods. Reproduced with permission from Ref. [17]. Copyright 2021 Elsevier.

Every year, more than 1748 million tonnes of fruit and vegetable waste (FVW) are produced worldwide [8]. Due to rapid economic growth, FW generation in Asia is predicted to increase by 278 to 416 million tonnes [9], resulting in an increase in world anthropogenic (carbon) emissions [10]. Malaysia is expected to produce 6.7 million tonnes of FW per year by 2020 [11]. In the EU, households account for over half of all FW [12]. Around 12 percent of the total EU food production is wasted in the United Kingdom [13], while the United States recorded the highest FW rates per capita at 278 kg [14]. FVW is a major component of FW, particularly in industrialized countries [15,16]. Figure 1 shows the FW management practices and the novel or emerging valorization approaches [17].

**Figure 2:**
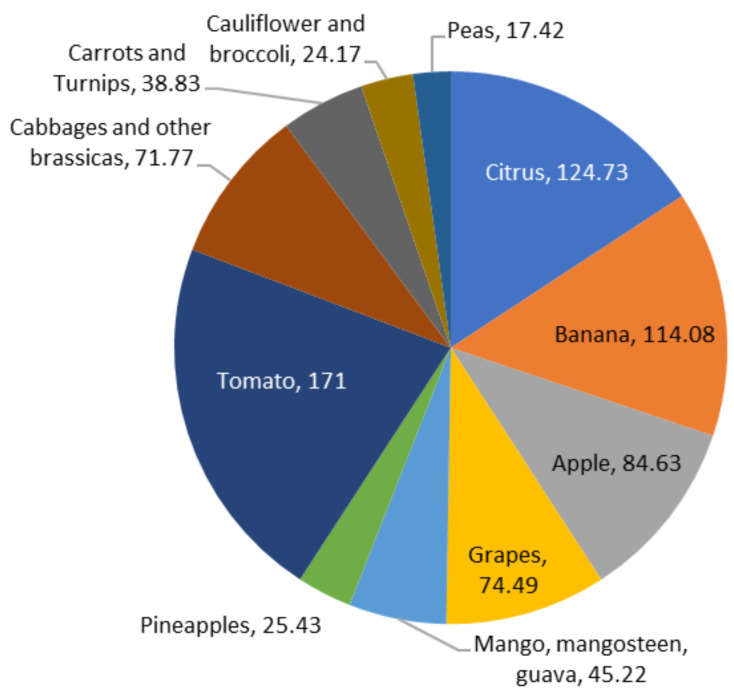
A significant amount of fruits and vegetables (in MMT) are produced globally.

According to Fabi et al. [18], median losses are estimated to be higher than 10 percent in Africa and Latin America for fruits and vegetables. In comparison, they range between 4 and 7 percent in Europe and North America. Figure 2 depicts the amounts of fruits produced globally, including 124.73 million metric tonnes (MMT) of citrus, 114.08 MMT of bananas, 84.63 MMT of apples, 74.49 MMT of grapes, 45.22 MMT of mangoes, mangosteens, and guavas, and 25.43 MMT of pineapples. Besides the production of potatoes, which was recorded at a considerable 3820.00 MMT, other vegetables are also shown in Figure 2, including tomatoes (171.00 MMT), cabbages and other brassicas (71.77 MMT), carrots and turnips (38.83 MMT), cauliflowers, broccolis (24.17 MMT), and peas (17.42 MMT) [19]. Due to losses in sales and transportation costs, such wastage, including subsequent generation of FVW, raises market operation costs, resulting in higher inflation.

The dairy, meat, fishery, and seafood processing sectors are the primary contributors of trash from animal wastes. Numerous types of residues can be identified from vegetable wastes, including grains, roots and tubers, oil crops and pulses, and fruits and vegetables, depending on the source [20]. Typically, FVW is characterized by a high water content and a high concentration of biodegradable organic substances (e.g., carbohydrates, lipids, and organic acids) [21]. As a result of these varied organic wastes, typical solid waste management systems (such as landfills and incineration) may cause serious environmental impacts, such as the discharge of leachate and the production of greenhouse gases [22]. 

**Figure 3:**
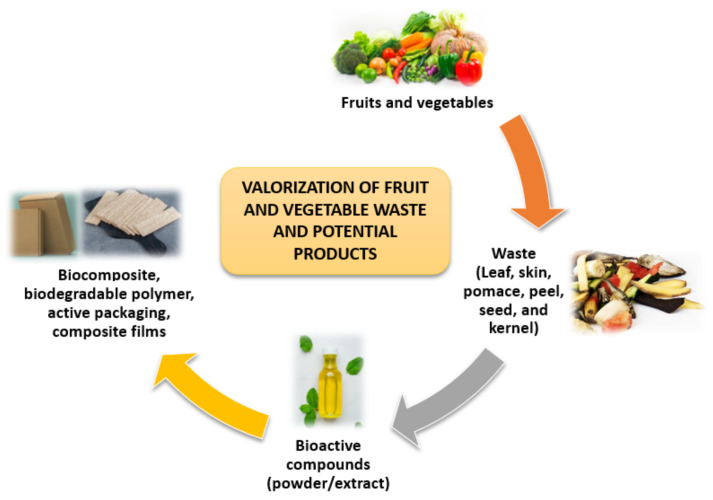
Valorization of FVW and potential products.

FVW is generated in large quantities in open markets, but little information is available on the actual volume of waste generated. Since significant amounts of waste are produced by the fruit and vegetable value chains, biorefinery concepts have been developed to valorize these wastes [23]. Waste from the FVW can be reused or recycled, which is more environmentally beneficial than disposal through open dumpsites or incineration [24]. Wholesale marketplaces, supermarkets, and agricultural activities are the traditional sources of FVW. Fruits and vegetables produce FVW at every stage of the chain, including production, transportation, storage, distribution, and consumption [25]. The valorization of FVW and potential products are shown in Figure 3.

There are several reviews on the applications of fruit and vegetable wastes. A review conducted by Bayram et al. [26] focused on the potential applications of fruit- and vegetable-based by-products such as biopolymers, biocomposites, active or intelligent packaging, and edible films and coatings. The authors discussed the advantages, disadvantages, and applications of these by-products as well. Kadzińska et al. [27] reviewed the role of specific chemical compounds found in fruits and vegetables, particularly in designing the physicochemical and functional properties of edible packaging materials. The advantages and disadvantages of using fruit and vegetables as components in matrix-forming solutions and their potential applications, future trends, and issues to consider when commercializing these products were discussed within the context of sustainable development.

In the fruit and vegetable processing industry, by-products such as peels, seeds, and shells are produced in large quantities [28,29]. These by-products contain high concentration of bioactive components such as antioxidants (polyphenols and dietary fibers), pigments and flavor compounds, proteins, essential oils, enzymes, and dietary fibers [30]. Coman et al. [31] looked at the bioactive potential of fruit and vegetable by-products and how they could be used in the food industry (functional foods) or the health sector (nutraceuticals). Several applications of food vegetable waste incorporated into the meat and its derivatives were reviewed by Calderón-Oliver and López-Hernández [32]. The peels and seeds of fruits, such as grapes, pomegranates, avocados, and citrus, are the most commonly used vegetable by-products because they help inhibit oxidation (lipid and protein) and the growth of pathogenic and deteriorating bacteria in the food supply. Adding these by-products to meat products can sometimes improve the quality of the product while also extending its shelf life. In food processing of waste and biomass by-products, pectin is one of the most common constituents; therefore, improving pectin extraction and recovery is critical to completely valorize these significant feedstock resources [29]. As a result, it is crucial to investigate the composting process of FW, particularly FVW, within this framework.

Given the potential for the valorization of such organic waste, research on the proper use of waste materials obtained from horticulture commodities may generate sustainable development initiatives to minimize environmental problems [19]. Significant products can be produced from these organic wastes, including active packaging, biopolymers, biocomposites, and other by-products. This review also explores the innovative technology (thermal and non-thermal) for bioactive compounds extraction based on the natural resources from fruit and vegetable losses and waste.

## 2. Active Packaging and pH Indicator Film

### 2.1. Active Packaging

The integration of additives into the packaging system to maintain or enhance the quality of food products and shelf life is referred to as active packaging [33]. Active packaging concepts include moisture absorbers, gas scavengers, carbon dioxide emitters, antioxidant, and antimicrobial-releasing and-containing systems. For example, the addition of antimicrobial additives into the packaging materials can minimize the risk of food spoilage and contamination from microorganisms [34]. Several additives have been successfully incorporated into packaging materials, including organic acids and their salts, bacteriocins, enzymes, chelators, and a range of plant extracts [35,36,37]. Due to concern about the health hazards of synthetic additives, researchers have been implementing various natural plant extracts to the biopolymers as active components [38]. In addition, active ingredients from FVW have added another level to active packaging systems as a solution for reducing FW. Several studies have focused on utilizing these wastes to make active films and investigated their properties, as summarized in Table 1.

For example, Luchese et al. [39] added blueberry pomace into cassava starch films. The aromatic compounds in blueberry pomace improved the light barrier properties, indicating the films’ ability to preserve food against UV lights. At the same time, the films were structurally stable when immersed in water for more than 24 h. The authors suggested the feasibility of the films for packaging aqueous food products. 

In another study, fiber and ethanolic extracts from blueberry juice from processing waste were used to make active films from gelatin capsules wastes [40]. The study found that the films with fiber showed reduced tensile strength and increased water vapor permeability with improved light barrier activity against UV light, which effectively reduced the lipid oxidation of sunflower oil. Both films showed significant decreases in light transmission and stability in antioxidant activity over 28 days.

Ali et al. [34] utilized pomegranate peel as a filler and an antimicrobial agent in developing films with hydroxypropyl high-amylose starch. The results showed that the tensile properties of the films improved and the pomegranate peel inhibited the growth of both Gram-positive (*S. aureus*) and Gram-negative (*Salmonella*) bacteria. On the other hand, research done by dos Santos Caetano et al. [47] produced biodegradable films based on minimally processed pumpkin residue extract (PRE) (0 to 6%) incorporated with cassava starch, glycerol, and oregano essential oil (OEO) (0 to 2%). The addition of pumpkin residue is crucial, since it provides opacity to the films, but it was not effective in improving the antioxidant and antimicrobial properties. 

Other researchers also used pomegranate peel as an antibacterial additive with sodium caseinate powder [48]. The peel resulted in a decrease in tensile strength and increased water vapor permeability (WVP). The films showed profound growth inhibition for Gram-positive (*S. aureus*) rather than Gram-negative (*E. coli*) bacteria. Shukor et al. [41] prepared tapioca-starch-based films incorporating thymol, jackfruit skin, and straw. Improvement in mechanical and barrier properties was observed for the films. The incorporation of skin and thymol retarded bacterial and fungal growth due to the antimicrobial activity of the films.

### 2.2. pH Indicator Films

Researchers are currently attracted to intelligent packaging systems, whereby active ingredients such as dyes and pigments are added into the film as pH indicators to trace and monitor food freshness throughout the storage period. This is related to the interactions between the food and its environment and the packaging material [49]. Table 2 shows some of the fruit and vegetables used as pH indicators in packaging films.

The colorimetric pH indicator films display apparent color changes with alterations of the food pH due to food deterioration and extrinsic environmental changes [53]; thus, from these color changes, the consumers receive authentic information regarding the food’s quality and its edibility, which is not achievable from the expiry date written on the package. As a pH indicator dye replacing synthetic pigments, natural dyes are now used mainly in biodegradable packaging materials [54,55]. Interestingly, the natural dyes and pigments from waste and by-products from fruits and vegetables have also been implemented as pH sensing dyes into packaging films to ensure food safety [56]. 

A study was conducted by developing colorimetric indicator film from mulberry based on gelatin and polyvinyl alcohol (PVA), whereby anthocyanin extract from the residue of mulberry processing was incorporated. This study showed that the mechanical properties were improved and visible color changes were shown when monitoring fish spoilage [57]. In addition, an earlier study produced films from cassava starch and blueberry residue that were rich in anthocyanin. Insertion of blueberry residue produced less compact films with high oxygen permeability. The films exhibited visual color changes in the pH range of 2 to 12 [56]. Luchese et al. [50] developed biodegradable pH indicator films based on cassava starch, utilizing the blueberry residue obtained after juice processing. The researchers deliberated on two different particle sizes for the blueberry residue powder for film preparation. They found that the films with smaller particles were more uniform and homogenous in appearance and the color change with pH was more intense than films with large particles. 

In addition, the tensile strength of the films decreased whereas elongation increased; simultaneously, the water vapor permeability of the films increased due to the presence of the particles and their heterogeneity. In another study, blueberry agro-industrial waste, a co-product from juice processing, was successfully used to develop pH indicator films based on corn starch [51]. The films changed their colors in response to different pH, and the color changes were visually perceptible to the human eye.

**Figure 4:**
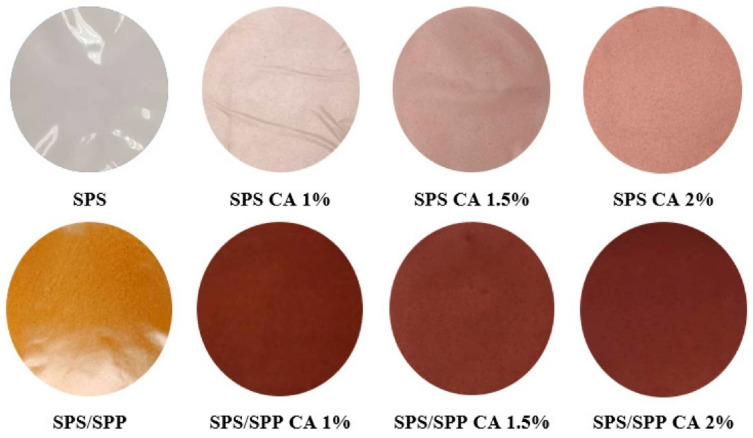
Visual appearance of SPS and SPS/SPP films at CA of 0, 1, 1.5, and 2%. Reproduced with permission from Ref. [52]. Copyright 2021 Taylor & Francis.

Black chokeberry (*Aronia melanocarpa*) pomace extract (a residue material after juice pressing) was chosen as a pH sensing dye of chitosan films. The addition of pomace extract reduced the solubility and swelling of chitosan, and these indicator films maintained integrity in acidic pH environments [58]. Sohany et al. [52] utilized sweet potato peel powder (SPP) as a filler to develop sweet potato starch (SPS)-based pH indicator films incorporating purple sweet potato anthocyanin. The films with peel exhibited reduced tensile strength with higher swelling and WVP values. Visually, the films were dark maroon and changed their color in response to various pH buffers, as shown in Figure 4. The films successfully indicated chicken freshness by changing their color with changes in pH of the deteriorated chicken.

## 3. Biocomposites

Several biocomposites products are produced from agro-industrial wastes whereby the fibs, proteins, carbohydrates, organic acids, and oils are extracted from the wastes, followed by fermentation and enzymatic processing [59]. Biocomposites produced from natural fillers/fibers and biodegradable plastics are examples of biodegradable materials. Starch can be obtained through the extraction process [60], whereas polylactic acid (PLA), polybutylene succinate (PBS), and polyethylene (PE) are produced by fermenting, chemical processing, and polymerizing their monomers [61,62]. The polyhydroxyalkanoate (PHAs) are synthesized by bacterial fermentation [63]. The carboxymethyl cellulose (CMC) can be produced via etherification [64]. Starches are partially modified to make biopolymer; PLA is widely used in food packaging; PHAs used for water-resistant packaging and injection molding [65].

Further, the peel/skin, seeds, and pomace of fruits and vegetables contain fibers used as filler in the film matrix to improve the biopolymer properties [39,52]. Pectin is another compound extracted from wastes and used in the polymeric matrix [35]. Also, peel and seeds contain more phenolic compounds than their fleshy parts [26]. For example, mango peel has a high level of phenolic content compared to its flesh [66]. The presence of phytochemicals and bioactive compounds provide preservative effects such as antimicrobial activity with anti-inflammatory and antioxidant attributes [67]. The blending of additives to polymers is found to improve the mechanical and barrier properties. Depending on the presence of additives, the polymers also function as pH indicators (sensor) or antimicrobial films [34,39]. Moreover, abundant organic wastes are available in skin and pulp, including citrus fruits such as orange, grapefruit, pineapple, mandarin/tangerine, lemon, and lime; seed waste from mango, grape, and pumpkin; skin from potato, sweet potato, jackfruit, pomegranate, and banana [65]. The utilization of these wastes into packing materials offers an essential alternative to conventional plastic packaging and contributes to a sustainable environment.

Despite substantial research on natural fiber composites, little is known about incorporating FW into biocomposites. Most biocomposites from FW research have focused on the biomass such as olive, pineapple, and banana. The mechanical and thermal properties of composites made from these biomasses and from other fruits are thoroughly investigated. Fiber treatment, type of polymer matrix, amount of fiber, compatibilizer, and process techniques have significant impact on the properties of biocomposites. Table 3 summarizes the residues from fruits and vegetable waste used in the production of biocomposites. 

### 3.1. Lignocellulosic Fiber

Fibers extracted from agricultural residues such as fruit and vegetable waste, woodland residues, and farming deposits are rich in cellulose, hemicellulose, lignin and are termed lignocellulose. These lignocellulosic fibers are obtained from biosources such as bast, foliage, fruit, kernel, timber, farmed excess, lawn, etc. Natural fibers possess comparable or even better mechanical properties like glass or aramid fibers [82]. Nanocomposite films based on lemon waste, 3% cellulose nanofiber (CNF), and 3% savory essential oil (SEO) are fabricated and are shown to enhance the barrier and mechanical properties. Film from lemon waste showed antibacterial properties against five foodborne pathogens [83].

Szymańska-Chargot et al. [36] evaluated the mechanical, hydrophilic, thermal, and antibacterial properties of nanocomposite made of PLA and nanocellulose. The nanocellulose is a carrot CCNF isolated from carrot pomace modified with silver nanoparticles. The nanocellulose modified with metal nanoparticles at a concentration of 0.25 and 2 mM was prepared earlier before combining with PLA. Composite containing CCNF with 2 mM of AgNPs showed the most significant improvement in mechanical properties. The degradation temperature was lower for PLA with CCNF/AgNPs, and this addition also increased hydrophilicity. The addition also improved transmission rates of oxygen, nitrogen, and carbon dioxide. It also acquired antibacterial function against *Escherichia coli* and *Bacillus cereus*, suggesting the lack of migration of nanoparticles from the composite.

Mohd Nordin et al. [68] studied the effect of freeze-dried durian skin nanofiber on the physical properties of PLA biocomposites. Durian skin nanofiber (DSNF) was developed using a freeze-drying (FD) process from durian skin fiber (DSF), and cinnamon essential oil was added as a plasticizer for PLA biocomposites. The tensile strength of these composites showed significant changes in the presence of DSF and DSNF in PLA.

**Figure 5:**
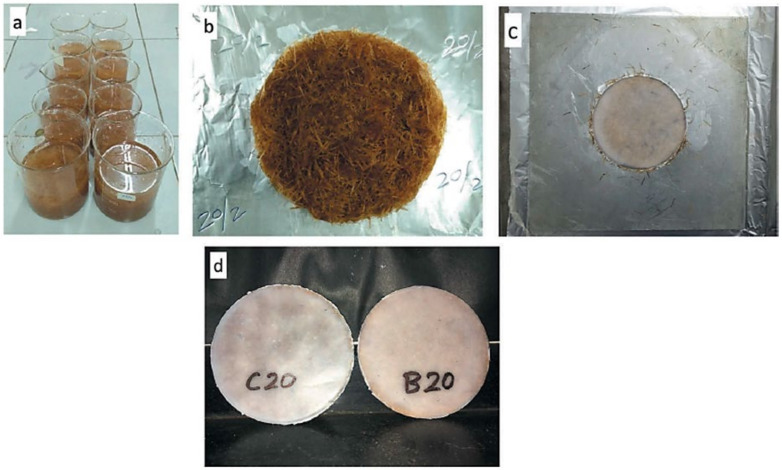
The process includes (**a**) fiber treatment followed by layering of fibers to form (**b**) fibrous bed, composite sample preparation through (**c**) compression molding, and (**d**) final composite samples. Reproduced with permission from Ref. [69]. Copyright 2021 Taylor & Francis.

Fibers from bananas have the potential to be incorporated into sound insulation composites. Singh and Mukhopadhyay [69] studied the effect of hybridization on sound insulation of coir-banana-PE hybrid biocomposites. These were prepared as shown in Figure 5 with chopped and randomly oriented coir and banana fibers. PP was used as a matrix, and a compression molding technique for composite fabrication. Hybrid and nonhybrid composites from coir and banana fibers were prepared at total fiber loading of 5, 10, 15, 20, and 25% by volume. The ratio of both fibers in hybrid biocomposites was maintained at 1:1. It was found that an increase in fiber loading considerably improved sound insulation up to a certain limit. The difference in transmission loss at the minimum and maximum fiber loadings for nonhybrid was higher for the finer banana fibers.

Anuar et al. [70] developed durian skin fiber (DSF)-reinforced PLA biocomposites with the addition of epoxidized palm oil (EPO). The amount of energy required for the production of the biocomposites was studied. The results showed that the PLA/DSF/EPO biocomposites had lower negative impacts as compared to the PLA/DSF biocomposites because the EPO improved the workability and processability of the biocomposites. They concluded that the plasticized PLA/DSF biocomposites could be potential biodegradable food packaging materials, as they have acceptable properties and produce no waste.

Gisan et al. [71] investigated the tensile, water absorption, and biodegradation properties of PLA/durian husk fiber (DHF) biofilms. The PLA/DHF biofilms with different DHF contents (0, 5, 10, 15, and 20 wt.%) were prepared via a simple solvent casting method. The results revealed that the tensile strength and modulus of elasticity of the biofilms increased with increasing DHF content from 5 wt.% to 10 wt.%. The tensile strength and modulus of elasticity of the PLA/DHF biofilms decreased compared to the neat PLA film due to the plasticized effect in the biofilms; however, the enzymatic degradation with α-amylase and the water absorption properties of the PLA/DHF biofilms increased with the DHF content.

Sea mango (SM) fiber was used as a filler in PP polymer biocomposites. Ong et al. [72] investigated the flexural and thermal properties of 5 to 25 weight percentages of SM added into the PP. The results showed an improvement in the flexural strength and stiffness when the SM content increased. The thermal stability and degree of crystallinity results were positive when a compatibilizer (such as PP-g-MA) was incorporated into the biocomposites.

### 3.2. Extract

Several natural extracts have been used as active additives to develop antioxidant-enriched films for food packaging applications. Natural antioxidants of plant extracts (PE) derived from various non-edible portions of fruit and vegetable by-products, such as peels, stones, and seed extracts, often contain a high amount of phenolic substances [84] and have been used as active ingredients in the manufacture of active films.

The valorization of fruit pomace (chokeberry, blackcurrant, apple, and raspberry pomace) in biocomposites was achieved by Żelaziński [73]. The mechanical and physicochemical characteristics were studied. The results showed that adding 30% chokeberry, apple, raspberry, and currant pomace substantially contributed to the improvements in flexural strength (between 11.1 and 12.3 MPa) and the increase of the water contact angle of the surface by 40%.

Tanwar et al. [74] investigated the characteristics of PVA starch incorporated with coconut shell extract and sepiolite clay as an antioxidant film for active food packaging applications. An active antioxidant film was fabricated using polyvinyl alcohol (PVA) and corn starch (ST) and incorporated with 3, 5, 10, or 20% (*v*/*v*) coconut shell extract (CSE) and sepiolite clay (SP) for the first time. It was found that the addition of CSE to films enhanced their antioxidant activity properties by up to 80%. Further, increasing the amount of CSE resulted in color changes in the active films and improved their thermal properties.

Rangaraj et al. [75] investigated the effects of date fruit syrup waste extract (DSWE) on the physical properties of gelatin films. The results showed that the loading of DSWE did not affect the thickness of the material. The moisture content and water solubility, on the other hand, increased with an increase in DSWE from 5 to 25 wt.%. PE/sour cherry shell powder biocomposite was investigated as a potential food packaging by Farhadi and Javanmard [80]. The addition of 2.5% sour cherry shell increased the elastic modulus, tensile strength, and mechanical properties of the composite. The increased sour cherry shell powder loading from 2.5 to 7.5 wt.% increased the moisture absorption and water vapor transmission.

Grapefruit and pomelo are commonly consumed fruits with higher levels of essential oils in the peels than other fruits [85]. Previous studies have reported that 10% extract of *Citrus paradisi* (grapefruit) peel obtained by microwave-assisted extraction (MAE) incorporated with multilayer low-density polyethylene (LDPE)/polyethylene terephthalate (PET) showed high antioxidant levels and acts as a free radical scavenger [86]. Pumpkins seeds and peels are the waste types generated from the processing industry, with high potential for utilization as biodegradable films. Defatted pumpkin seeds (DPS) and pumpkins peels (PP) with glycerol and lecithin are produced in the process. The films exhibited the highest tensile strength values (1401 ± 5.4 kPa) and good elongation (9.74 ± 0.46%). The properties of the films were improved using ultrasound treatment. Generally, waste from the pumpkin processing industry is successfully developed as biodegradable films [87]. 

### 3.3. Powder and Husk

The incorporation of pomegranate (PMG), papaya (PPY), and jackfruit (JF) peel into gelatin/PE bilayer films led to significant increases in thickness, opacity, and moisture content (*p* < 0.05) but reduced film solubility in water. Films incorporated with pomegranate (PMG) exhibited high antimicrobial and antioxidant properties [88]. 

Mardijanti et al. [76] examined the material characteristics of cocopith and evaluated the potential of a mycelium-based biocomposite as an insulator. Dry cocopith, which is the residue from the coconut coir milling industry, was mixed with wood powder, pollard (bran), lime, tapioca, and Ganoderma mushroom seeds and then put into baglogs. The solid baglogs were then removed from the molds, dried, and compacted to the desired size using a hot press. The potential as an insulator was validated via a thermal conductivity test at temperatures of 13 to 40 °C. The test showed a thermal conductivity value range of 0.0887241 to 0.002964 W/mK. A value ranges of 0.01 to 1.00 W/mK is recommended for thermal conductivity insulators.

A study on the enhancement of the mechanical behavior of a PLA matrix using tamarind and date seed micro fillers was conducted by Nagarjun et al. [77]. The composites were manufactured using the compression molding technique. The tensile results showed that the seed filler reinforcement significantly improved the tensile strength of the PLA matrix. The maximum tensile strength values achieved with TI/PLA and PD/PLA were 72.42 MPa and 61.39 MPa, respectively. The particulate reinforcements of both tamarind and date almost doubled the flexural and impact strengths of the PLA matrix. Moreover, the date seed powder-incorporated composites showed a 34.68% improvement in microhardness. The uniform dispersion of the filler was evident in TI/PLA and PD/PLA with 2 wt.% filler, which contributed to their better tensile strength. Conversely, increasing the filler content to 4 wt.% resulted in agglomeration of the fillers and subsequently contributed to the low mechanical strength of the composites.

Reinaldo et al. [78] investigated the effects of grape and acerola residues on the antioxidant, physicochemical, and mechanical properties of cassava starch biocomposites. Various concentrations of grape skin (Gr) and acerola (Ac) residues (0.1, 1.0, 5.0, and 10.0 wt.%) were prepared using extrusion and injection molding processes. The large size distribution of cassava starch may favor the plasticization stage to obtain TPS compared to the different types of starch obtained from other plant sources. The results showed that the addition of grape skins and acerola residues to the cassava thermoplastic starch resulted in better antioxidant characteristics. The addition of grape residue in TPS resulted in decreased in elongation at the break compared with pure TPS, which was more significant with higher concentrations of grape residue (5.0 and 10.0 wt.% of Gr). The similar mechanical behavior was recorded by Gutiérrez et al. [89] and Deng and Zhao [90].

**Figure 6:**
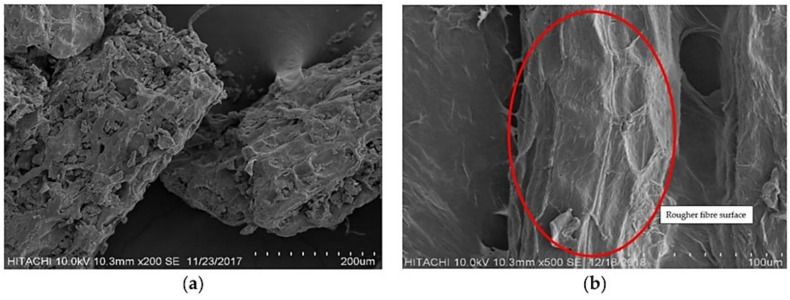
Scanning electron microscopy (SEM) micrographs of (**a**) unbleached jackfruit skin powder (JSP) and (**b**) bleached jackfruit skin powder (BJSP). Reproduced with permission from Ref. [79]. Copyright 2021 Elsevier.

Marzuki et al. [79] studied the effects of jackfruit skin powder (JSP) and fiber bleaching treatment in PLA composites incorporating thymol. The insertion of 30 wt.% jackfruits fibers gave the best tensile performance. The elongation at the break decreased with increased fiber loading, regardless of treatment, but no significant changes from 10 to 30 wt.% loading of powder were observed. All JSP or bleached jackfruit skin fiber (BJSP) composites showed higher tensile modulus than pure PLA, and the results were in agreement with Suradi et al. [91]. The SEM micrographs in Figure 6 show the fiber surface differences between unbleached JSP and BJSP, respectively. Following bleaching treatment, a rougher fiber surface is shown, indicating effective removal of the non-cellulosic components’ potential for good mechanical fiber locking with the matrix.

Torres et al. [81] evaluated the influence of chestnut husk content on the mechanical properties of novel starch/chestnut husk biocomposites. This was developed by incorporating 2.5, 5, and 7.5 wt.% chestnut husks via an extrusion molding procedure. The results showed that the pure starch samples had an average elastic modulus of 3.30 MPa, while starch samples with 7.5 wt.% chestnut husks displayed an average elastic modulus of 4.85 MPa. The ultimate tensile strength value was independent of the chestnut husk content, while the elongation at the break point decreased as the filler content increased. These results were in agreement with those of previous reports on starch-based biocomposites reinforced with cellulosic fillers such as cotton, hemp, and winceyette fibers [92,93,94].

Othman et al. [43] developed tapioca-starch-based biodegradable film incorporating banana pseudostems (waste) powder for the starch-based films. The mechanical and optical properties of the films were reduced but the barrier activity improved. The optimum percentage composition of 40 wt.% pseudostem powder can be used for incorporation into starch-based films as food packaging.

Cassava starch-based films have been investigated by Leites et al. [95] to determine the effect of waste from the production of orange juice on the properties of the films. The orange waste was added in two different forms, which are powder and aqueous extract (by soaking the powder in water followed by filtration). When comparing the moisture content, water solubility, and thickness of the extract to that of the powder orange residue, it was discovered that the extract had higher values.

### 3.4. Isolation of Fiber from FVW

For the extraction of cellulose from carrot waste, polysaccharides were extracted in acidic and alkaline environments before being treated with sodium hypochlorite solution to remove lignin and other lignin-containing compounds. With this treatment method, carrot cellulose was obtained as a 4% concentration in water suspension after the treatment. The nanocellulose was created by homogenizing cellulose with ultrasonically homogenized cellulose. Because the sonication system included a temperature probe, an ice bath was used to keep the samples from becoming too hot. The amplitude of the ultrasonic homogenizer was maintained at 90% of its maximum. Finally, carrot CCNF samples containing 0.1 wt.% carrots were obtained [36].

The extraction of fiber from durian skin required only a few simple procedures. The durian skin was cut into smaller pieces and thoroughly washed with tap water to remove any dust or adhering particles before being prepared. Next, the skin was dried at 70 °C for 24 h. The dried skin was ground to obtain fibers ranging in length from 100 to 150 μm. Approximately 300 g of raw durian skin fibers was used in this process. The alkali treatment of DSF was carried out with the help of sodium hydroxide (NaOH). [70].

The chemical treatment was used to isolate the orange peel. Eight grams was kept at room temperature under mechanical stirring for 16 h in a 5% KOH solution. Following the alkaline treatment, the insoluble residue was bleached with NaClO_2_ solution for one hour at 70 °C, pH 5.0, which was adjusted with 10% acetic acid. The residue was neutralized, washed, and centrifuged at 6000 rpm for 20 min at 25 °C. The second alkaline treatment was repeated with a concentration of 5% KOH. The insoluble residue was subjected to acid hydrolysis for an hour at 80 °C with 1% H_2_SO_4_ for one hour. Following centrifugation and washing of the final residue, the diluted suspension was stored at 4 °C in a sealed container. The cellulose suspension was dried by lyophilization and stored at 4 °C, where it was designated as NFC [96].

The cellulose fiber from banana peels was obtained by removing the hemicellulose and lignin. The banana peel was treated with 10% (*w*/*v*) natural lye, which was made by soaking wood charcoal ash in water for 48 h before being applied. The extracted cellulose fiber was then bleached twice with 10% (*v*/*v*) hydrogen peroxide at 90 °C for 10 min each time. As previously described, the white cellulose fiber was retreated with a lye solution. Finally, cellulose was obtained [97].

## 4. By-Products

Fruits and vegetables are crucial for human nutrition, providing significant amounts of daily vitamin, mineral, and fiber needs. The fruit and vegetable processing industries generate significant amounts of waste in the form of liquid and solid, which contain several reusable high-value substances with significant economic potentials. Fruit and vegetable by-products accumulated from industrial operations. such as bagasse, peels, trimmings, stems, shells, bran, and seeds, make up more than half of all fresh fruit and have nutritional and functional contents that are sometimes higher than the finished product [98]. These by-products can be applied in food and other non-food applications, including medical, pharmaceutical, energy, and chemical production.

Typically, the fruit processing activities generate solid wastes (peel, skin, pulp, pomace, seeds, bunch, stems, and shells) and liquid wastes (crude extracts and wash water). With proper management, these wastes can be utilized for the production of by-products. Efforts have been made to explore the potential of these by-products in many applications, including in food and non-food industries. 

**Figure 7:**
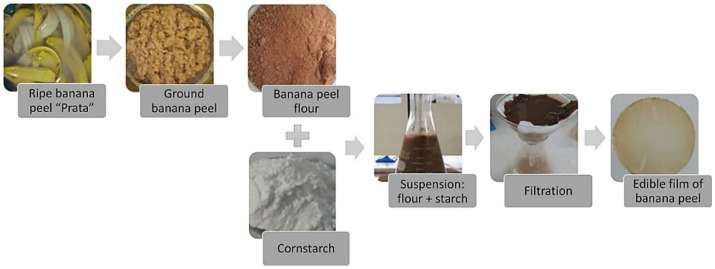
Development of edible coatings from banana peel. Reproduced with permission from Ref. [100]. Copyright 2021 Elsevier.

Based on recent literature, most of the progress on utilizing fruit waste has been towards food and polymer applications, particularly in the production of flour, fiber, and pectin as fillers. Khoozani et al. [99] used banana pulps and peels to produce flour by varying the drying process using oven drying, a spouted bed drier, ultrasound, a pulsed vacuum oven, a microwave, spray drying, and lyophilization. Additionally, flour made from ripe “Prata” banana (*Musa* spp.) peels was used in the development of edible coatings, as shown in Figure 7 [100]. 

Reißner et al. [101] processed the pomace of different berries (blackcurrant, redcurrant, chokeberry, rowanberry, and gooseberry) into flour and fruit powder. de Andrade et al. [102] used orange, passion fruit, and watermelon to produce flour. The prebiotic potential of the fruit-based by-product flour obtained from the solid waste from fruit processing was evaluated after undergoing in vitro gastrointestinal digestion process.

In addition to flour, fiber is another food application for fruit waste by-products. Sharma et al. [103] processed apple pomace to produce fiber to prepare fiber-enriched products. The dried pomace was packed in gunny and PE bags and stored at low (0–4 °C) and ambient temperatures (13–26 °C) after washing, blanching, and drying in a polytunnel drier (45 ± 8 °C). The apple fiber can be used in product formulations with good cholesterol-lowering characteristics and for the establishment of polymer composites such as reinforcements with epoxy resin to form hybrid composites [104].

Begum and Deka [105] used banana bract to extract dietary fiber (DF) using an ultrasound-assisted extraction method combined with alkaline extraction. This antioxidant DF is vital because it is linked to various health benefits, including preventing and treating chronic and degenerative diseases. Additionally, Mir et al. [106] produced gluten-free crackers with high fiber from brown rice flour and apple pomace. Apple pomace flour blends were made by combining brown rice flour with 0, 3, 6, and 9% apple pomace.

**Figure 8:**
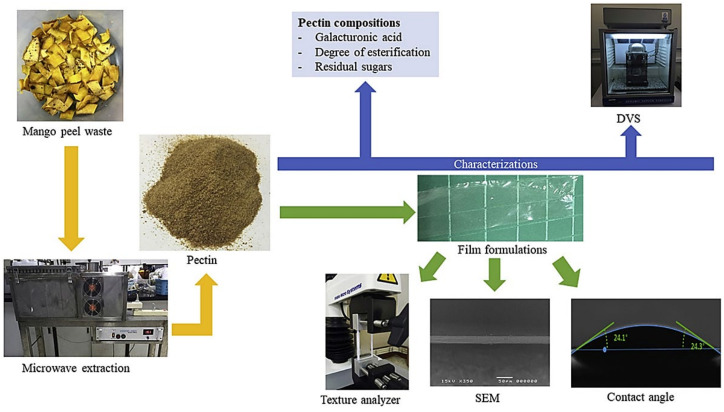
Fabrication of thin polymer films from mango peel waste. Reproduced with permission from Ref. [112]. Copyright 2021 Elsevier.

Another common use of fruit waste is that of pectic substances for use in other applications. Khamsucharit et al. [107] extracted pectin from the peels of five banana varieties through a conventional hot acid extraction method with a citric acid solution. Another study on pectin extract from banana peel was conducted by Maran et al. [108] using an ultrasound-assisted, citric-acid-mediated extraction method, optimized through the response surface methodology (RSM) approach. Moorthy et al. [109] used jackfruit peels to produce pectin using an ultrasound-assisted extraction method, a similar approach to that used by Hosseini et al. [110] and Guandalini et al. [111], who respectively extracted pectin from orange and mango peels. Chaiwarit et al. [112] found that pectin extracted from mango peel (Figure 8) can be regarded as a potential biopolymer for an edible film for food packaging.

**Figure 9:**
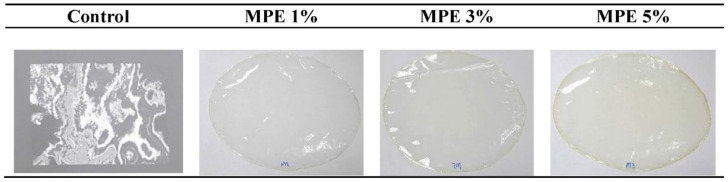
Gelatin films with various concentrations of mango peel extracts. Reproduced with permission from Ref. [114]. Copyright 2021 Elsevier.

Fruit waste has also been used for the production of non-food by-products. One of the most common applications of fruit waste is for the production of bioadsorbents. Laysandra et al. [113] used durian skin to produce adsorbent by mixing it with acid-activated bentonite (AAB) and natural surfactant (rarasaponin). They found that the rarasaponin/bentonite-activated biochar from the durian shell composite (RBAB) was effective for use as a new composite adsorbent for the removal of crystal violet and Cr (VI) from aqueous solutions. Meanwhile, fruit waste is also utilized to make films. Mango peel was used in the production of fish-gelatin-based films [114]. The films were prepared via the solution casting method with three different concentrations of the mango peel extract (1 to 5%). The films produced were then tested for their physical, barrier, mechanical, and antioxidant qualities. Based on their findings, the addition of mango peel extract in the formulation produced a thicker, denser, and continuous structure with outstanding free radical scavenging activity (Figure 9). 

Fruit waste has also been used in the production of edible coatings to improve their properties. Blackberry pomace has reported been used for its the antioxidants and antimicrobials sources in the production of edible coatings for foods and consisting of carboxymethylcellulose, bacterial cellulose fibrils, and pectin sweets, with high lipid contents [115]. Meanwhile, grape seed extract has been included in pectin–pullulan edible film production for the storage of peanuts [116]. This coating successfully extended the shelf life of the stored peanuts by delaying rancidity.

Similar to fruit wastes, flour is the common by-product produced from vegetable waste. de Andrade et al. [102] used solid products from eight types of vegetables, including carrot, courgette, cucumber, lettuce, mint, rocket, spinach, and taro, to produce flour, then tested the modulatory effects on the gut microbiota composition. Amofa-Diatuo et al. [117] produced flour from cauliflower stems and leaves as a source of isothiocyanates (ITC) in an apple juice beverage. Cauliflower waste was also utilized in producing fermentable sugar. Majumdar et al. [118] pre-treated cauliflower stalks and leaves with dilute phosphoric acid prior to enzymatic hydrolysis to better release fermentable sugars. Vegetable waste is also a source of fiber and pectin. Iwassa et al. [119] produced and characterized fiber concentrates from asparagus by-products. They concluded that the fiber concentrates had high potential for use in formulating functional food products. Kazemi et al. [120] used an ultrasonic extraction method to extract pectin from eggplant peels. Eggplant peels provide substantial extraction yields and are considered to have high potential in pectin production

Waste from vegetables was also used for the extraction of essential oils. Chiboub et al. [121] extracted essential oils from the tops of carrots. The result showed that the essential oil produced was a good source of natural antimicrobials or aromatic agents. A similar finding was reported by Caliceti et al. [122] for peptide extract from cauliflower leaves. In contrast, the tops and roots of carrots were reported to be used to produce biodegradable composite films [123]. To improve the composite properties, they mixed and optimized the formulation with hydroxypropyl methylcellulose (HPMC) and high-pressure microfluidized cellulose fibers. Garlic peel extract was reported to be added in the formulated gelatin film as a source of antioxidant and antibacterial agents [124]. The gelatin film was proven to maintain the qualities of the rainbow trout fillets during refrigerated storage. Additionally, vegetable waste was also used for the production of the highly antioxidant edible coatings. Asparagus waste extract was incorporated into a hydroxyethyl cellulose–sodium alginate edible coating to significantly extend the postharvest life and retain the quality of strawberry fruit [125].

## 5. Innovative Technologies Used for Bioactive Compound Extraction

### 5.1. Thermal Extraction

Thermal extraction includes decoction, reflux, Soxhlet, hydro, and steam distillation techniques [126]. Incorporating bioactive compounds such as flavonoids, phenolic acids, and polysaccharides into polymer materials improves both the antioxidant and antibacterial activity [127]. Temperature increases the solubility and diffusivity in solid–liquid extraction, resulting in high yields of extracted compounds. Thermal degradation of enzymes and desired bioactive compounds occurs during prolonged extraction at high temperatures [128]. Regarding the extraction of FVW and FVB for industrial applications, various methods are used, such as fermentation and separation (lactic acid extraction from potato peels), hot dilute acid treatment and alcohol precipitation (pectin extraction from citrus peels), and steam distillation and alkali treatment (D-limonene extraction from citrus peels) [129]. These methods are appropriate for batch and large-scale processing. Meanwhile, emerging thermal processes, such as microwave-assisted extraction (MAE), pressurized liquid extraction (PLE), and subcritical water extraction (SWE), offer additional benefits and have the potential to replace conventional methods due to being efficient, economical, and environmentally friendly [17].

#### 5.1.1. Conventional Heat Extraction

One of the oldest extraction methods is conventional heating extraction (CHE). CHE involves direct contact with a thermal source either by conduction or convection as the heating mechanism. The CHE thermal source usually is an used oven, hot plate, or water bath, while acidic solutions are used as the agents. Recent research on the FVW and FVB extraction methods showed that CHE was not favored as compared to the new emerging extraction technologies, such as MAE [130,131,132] and ultrasonic-assisted extraction (UAE) [133,134,135]. Although the CHE method produced more bioactive compounds than the MAE method, such as pectin extracted from black carrot pomace [132] and lime peel [136], the CHE method has several drawbacks, including high energy requirements and long processing time, making it inefficient in industrial applications; however, the combination of CHE and other techniques has shown tremendous potential in FVW and FVB processing. High PLE [137] and UAE [133] are two technologies that can be integrated with CHE, which can increase the extracted yield by over 50%. Table 4 shows the optimal yields of bioactive compounds from FVW that have been achieved in recent years.

#### 5.1.2. Soxhlet Extraction

Another traditional thermal extraction method is Soxhlet extraction (SE), which involves high temperatures, long extraction times, and a large amount of solvent, meaning this method triggers environmental concerns. Similar to conventional heating extraction, the SE method also results in highly extracted bioactive compounds from FVW and FVB, including papaya [138], grape [139], and soapberry seed oil [140]. This classical extraction method has been compared to MAE [141,142,143] and UAE [144,145]. Table 5 shows the findings from the latest studies on SE methods using FVW. Throughout the experiments, Fernandez-Pastor et al. [142] showed that olive peel took 30–90 min to yield around 19 to 23% of bioactive compounds as compared to other studies that required longer times for the extraction technique (4 to 8 h). 

Sukatta et al. [146] studied the bioactivity levels and characterization of rambutan peel extract (RPE) and the feasibility of RPE as a bioactive compound for antimicrobial and antioxidant applications in whey protein isolate (WPI)/cellulose nanocrystal film. The RPE was extracted for 16 h in a Soxhlet extractor with 95% ethanol. Using the HPLC chromatogram, the main components of RPE were corilagin, ellagic acid, geraniin, and gallic acid. The main bioactive components were classified as phenolic compounds, which exhibited antioxidant and antimicrobial properties. 

As an alternative to existing synthetic packaging films, Jridi et al. [147] developed grey triggerfish skin gelatin films containing phenolic extracts from blood orange (Citrus sinensis) peel. The dried orange peel extract (DOPE) was obtained by extracting the dried orange peels via Soxhlet extraction using 300 mL of ethanol for 6 h. Similarly, fresh orange peel extract (FOPE) was obtained by extracting the fresh orange peels. It was found that the extraction yield of the DOPE was 31.2% (*w*/*w*), which was significantly higher than that of FOPE (25.3%).

#### 5.1.3. Microwave-Assisted Extraction

Microwave-assisted extraction (MAE) is a powerful alternative method for extracting bioactive compounds from FW. It is mainly used for extraction because it reduces solvent usage, energy consumption, extraction times, heating rates and increases extraction efficiency and selectivity, resulting in quality target products [148]. Many studies were conducted using MAE methods on FVW and FVB, including longan seed [149], tomato peel waste [150], apple pomace [151], banana peel [152], pitaya fruit peel [130,153], passion fruit peel [130], lemon peel, mandarin peel, and kiwi peel [154]; thus, MAE is mostly used to extract pectin from fruit waste. This approach has been used to extract high-quality pectin with biomasses such as mango peels [155], citrus mandarin peels [156], fig skin [157], orange peel, apple pomace, mango peel, carrot pulp [158], pumpkin biomass [148], and banana peels [159]. The study conducted by Zin et al. [160] showed that the highest microwave power used on the fruit waste (sour cherry pomace) was 700 W. Other biochemical compounds can also be obtained using this technique, such as antioxidants from black carrot pomace [161], pitaya peel [153], and mango seed kernels [162]; flavonoids from Jocote pomace [163]; anthocyanins from grape juice waste [164] and sour cherry pomace [165]; and essential oil from lemon peels waste [166]. Casas et al. [167] also showed the potential of cocoa butter produced from mango kernel butter by extracting discarded seed kernels. Table 6 shows the summary conditions used for optimal bioactive compound extraction in recent studies on FVW.

For optimum yield extraction of the targeted compound, focused microwave-assisted extraction is preferred to conventional or household microwave ovens, as the parameters, namely the pressure and temperature, can be monitored [163]. A previous study showed that the combination of MAE with other methods could help produce high-yield compounds. Sequential ultrasound-microwave assisted extraction (UMAE) of fig skin extract resulted in higher pectin yield ~14.0%, with citric acid used as the solvents. Brönsted acidic–ionic liquid-based ultrasound-microwave synergistic extraction (BUME) from pomelo peels achieved the highest pectin yield of 328.64 ± 4.19 mg/g with optimum conditions involving 10 mM [HO_3_S(CH_2_)_4_mim]HSO_4_ solvent, 15 min of extraction time, 360 W of microwave irradiation power, and 27 mL/g liquid–solid ratio compared as compared to MAE (210.39 ± 5.82 mg/g). 

Utama-Ang et al. [168] studied MAE of dried ginger and developed a rice-based edible film containing ginger extract. The optimal MAE conditions were determined to be 400 W microwave power and one minute extraction time. At high temperatures and microwave power, 6-gingerol dehydrates water (H_2_O) from its structure, resulting in the formation of 6-shogaol4. Microwave power accelerated the retro-aldol reaction of 6-gingerol, and it is suggested that zingerone constituents be generated with aldehyde to deliver the products. The optimized extract showed good results in terms of the levels of total phenolic compounds (198.2 ± 0.7 mg GAE/g); antioxidant activity as measured by DPPH (91.4 ± 0.6% inhibition), ABTS (106.4 ± 3.1 mgTE/g), and FRAP (304.6 ± 5.5 mgTE/g); and bioactive compounds, including 6-gingerol (71.5 ± 3.6 mg/g), 6-shogaol (12.5 ± 1.0 mg/g), paradol (23.1 ± 1.1 mg/g), and zingerone (5.0 ± 0.3 mg/g).

In contrast, industrial potato peel by-products allowed greater antioxidant extraction yields than in combinations with ultrasound treatment [169]. Radiofrequency-assisted extraction (RFAE) with a frequency range of 1 to 300 MHz is another method used in dielectric heating as compared to microwave-assisted extraction (300 to 3000 MHz) in electromagnetic field-based thermal processes. An analysis discovered that the optimum conditions for RFAE were similar to MAE for pectin extraction from apple pomace. Still, the physicochemical properties (DE, GA, color values, and thermal stability) of apple pomace showed better RFAE pectin values [151].

#### 5.1.4. Pressurized Liquid Extraction

Pressurized liquid extraction (PLE) is a recent extraction method that has been widely used on seaweed, plants and food-related by-products [171], grape skin and seeds waste [172], passion fruit rinds [173], pomegranate peel [174], olive pomace [175], mulberry pulp [176], and beetroot waste [177]. Table 7 shows the optimum yield levels found in progress studies for the bioactive compounds found in FVW. The PLE method is becoming more popular due to its ability to keep the extraction parameters, temperature, and pressure steady. The solvent is heated at a high temperature and applied under high pressure to maintain the liquid conditions throughout the treatment [175]. PLE is a green technology technique that is employed to recover bioactive compounds and that has several benefits, such as reduced extraction time. It involves a significantly lower amount of organic solvent consumption due to its high mass transfer rate [173].

Cejudo-Bastante et al. [178] investigated the extraction process used in developing bioactive jute-fiber-based food packaging using pressurized liquid extraction (PLE) and enhanced solvent extraction (ESE) techniques. The extraction yield and antioxidant capacity levels of the red grape pomace extract (RGPE) obtained using ESE and PLE were compared under varying pressure (10 and 20 MPa), temperatures (55–70 °C), and co-solvent (C_2_H_5_OH or C_2_H_5_OH:H_2_O) conditions. They discovered that the PLE technique produced the most bioactive extract with 20 MPa, 55 °C, and one hour residence time using C_2_H_5_OH:H_2_O (1:1 *v*/*v*), providing antibacterial capacity against *Escherichia coli*, *Staphylococcus aureus*, and *Pseudomonas aeruginosa*.

#### 5.1.5. Subcritical Water Extraction

Compared to conventional extraction procedures, sub-critical water extraction (SWE) is a green extraction technology that yields superior quality extraction products and is cost-effective with a short extraction or treatment time [179,180,181,182]. SWE is also known as pressurized hot water or superheated water extraction, as it uses water at temperatures between 100 °C and 374 °C (critical temperature) and at pressures of up to 22.1 MPa (greater than vapor saturation) to keep the water molecules in a liquid state throughout the process [183]. Water is a polar solvent with a dielectric constant (ε) of 79.9 and a density of 1000 kg/m^3^ [184,185]. When water is heated to higher temperatures, its hydrogen bonds break down, resulting in a drop in its dielectric constant (ε), demonstrating water’s ability to act as a material reaction medium. Water has a density of 79.9% at ambient temperature and atmospheric pressure. Water may be lowered to 27–32.5% while remaining in liquid form by increasing the temperature to 250 °C and increasing the pressure to 5 MPa. Water has a similar density to methanol (32.5%) and ethanol (27%) at ambient temperature [186,187]. The latter enables water to interact with polar compounds, lowering the binding force and allowing substances to dissolve in water at greater temperatures and pressures.

The ionic constant of water (Kw) increases with increases in the reaction temperature according to Pourali et al. [188], and is nearly three times greater than at room temperature. Water’s reactivity increases as the concentrations of H^+^ and OH^−^ in the aqueous medium increase, causing it to act as an acid or base catalyst that is appropriate for hydrolysis reactions. Organic waste can, thus, be hydrolyzed and the necessary components contained inside can be removed using sub-critical water treatment [189]. Sub-critical water treatment is an environmentally favorable procedure because no chemical solvent is required. As a result, less effluent is created. Table 8 shows several examples of the application of SWE on FVW.

The extraction mechanism of SWE begins with solute desorption under elevated pressure and temperature, followed by the diffusion of extracted chemicals into the solvent. Finally, the extracted solutions are eluted from the extraction cell and transferred to a collection container [195,196].

Several process parameters influence the extraction efficiency of SWE, including the reaction temperature, pressure, reaction duration, solid-to-water ratio, sample particle size, pH, solute properties, and surfactant addition [197]; however, the reaction temperature, reaction duration, and solid-to-water ratio have the greatest influence on the SWE process. Because the viscosity and surface tension of the extraction solvent diminish with increasing temperature, Thani et al. [198] discovered that increasing the treatment temperature enhances the mass transfer rate and solubility of bioactive chemicals; however, if the temperature is raised above a certain point, the selected chemicals may degrade. As a result, the closely associated process temperature and duration should be optimized for each unique situation and are highly reliant on the desired product’s qualities. 

Ho et al. [199] investigated the influences of *P. palatiferum* freeze-dried powder (PFP) using SWE on the antioxidant activities and physical properties of gelatin–sodium alginate (GSA)-based films. *P. palatiferum* (Nees) Radlk. leaves were extracted with subcritical water, which increased the total phenolic content (TPC) and antioxidant activity as the PFP concentrations increased. The increase in antioxidant activity occurred in parallel with TPC. The antioxidant activity was attributed to the phenolic compounds [200] and gelatin–sodium alginate [201]. In addition to TPC, *P. palatiferum* leaves also contained a variety of other compounds, including protein, saponin, total sugar, and phytosterol [202], all of which can contribute to the antioxidant activity of GSA-based films.

Mohd Thani et al. [203] conducted an exhaustive review on SWE of sugar from FW. Monosaccharides and oligosaccharides are important carbohydrate molecules that can be hydrolyzed from FW. Sugar extraction from bakery waste, for example, is an effective way of valorizing this type of FW [198]. The leftover croissants had the most fructose and glucose at 4.74 and 3.76 mg/g substrate, respectively [198]; however, the sugar yield increased to a maximum value following SWE and then steadily decreased over time, whereas the yield of the degradation products increased over time [204,205].

Additionally, SWE can be successfully employed to extract antioxidant-rich extracts from yarrow by-products. The obtained extracts are rich in total phenols and flavonoids and have high antioxidant activity [206]. Oilseed cake extracts derived using subcritical water show a significant amount of promise for application in the fortification of various food goods and cosmetics. Depending on the type of oilseed, specific components such as the flavor amino acids aspartic acid, glutamic acid, and alanine can be extracted. This biowaste’s favorable chemical composition and high nutritional content provide it with high utilization potential [207]. The most significant yield of phenolic compounds (4855 mg/100 g dry weight) was obtained using subcritical water extraction from pomegranate seed remnants [208]. Similar results were obtained for phenolics in white wine grape pomace [209] and peach palm by-products [210].

### 5.2. Non-Thermal Extraction

Each non-thermal extraction process has its own set of advantages and disadvantages, as summarized in Table 9. Several factors must be considered to obtain the best results when extracting phenolic compounds from FVW. The operation’s success depends on the understanding of the nature of the target compounds, source materials, and waste matrices. Additionally, the process type and operating parameters used in the recovery process are critical determinants of the yield and quality of the recovered chemicals. The FVW matrix, the materials’ physicochemical properties, and the type of compounds extracted may affect the chosen approach. Conventional extraction processes with low yields require longer extraction times, large amounts of energy, and significant capital investment. Due to the difficulties associated with achieving high-purity target compounds, conventional technologies are regarded as inadequate compared to the emerging non-conventional approaches, such as ultrasound-assisted, HPP, and PEF.

When combined with a properly chosen extraction method, media, and optimized parameters, these technologies have been shown to increase the yield of specific chemicals from FVW while minimizing carbon footprints [220]. Although selective for lipophilic and volatile compounds such as fats and oils, certain methods such as SFE utilize CO_2_; thus, co-solvents are indicated to boost extract purity. As such, green extraction media should be thoroughly evaluated prior to extraction, especially when the desired chemicals are food-grade. In order to turn the FVW problem into a solution for recovering the valuable qualities of bioactive substances that are now being wasted, more research is required.

As such, this sub-section will focus on the innovative technologies used to extract bioactive compounds from FVW and will be limited to notable articles published in the previous decade.

#### 5.2.1. High-Pressure Processing

High-pressure processing (HPP) is commonly used to produce commercially available commodities such as minimally processed fruit juices, guacamole, jellies, dips, salsas, meat and poultry, seafood, and ready-to-eat meals [221,222]. Pasteurization at high pressures ranging from 400 to 600 MPa and temperatures ranging from 20 to 70 °C is a common industrial process. On the other hand, high-pressure sterilization at pressures greater than 600 MPa and temperatures ranging from 90 to 120 °C is more commonly used to eliminate resistant food enzymes, bacteria, and spoilage spores [223,224]. In addition, this technology is being researched for various applications, such as reducing allergenicity in meals, inactivating fruit and vegetable enzymes, and valorizing food matrices. High pressure benefits both fresh produce and FW by-products [225,226]. High-pressure extraction and infusion technologies, for example, may show promise for agricultural and FW valorization.

High-pressure treatment is a green method for extracting bioactive compounds from agricultural commodities. Flavonoids, polyphenols, ginsenosides, anthocyanins, lycopene, caffeine, salidroside, corilagin, and momordicosides are bioactive compounds that have varying polarity levels [226,227]. The steps in specific bioactive compound extraction methods are breaking down plant cell walls to free intracellular molecules, isolating the bioactive compounds from auxiliary components, and purifying them [228]. High-pressure extraction improves the bioactive and heat-sensitive chemical extraction processes while requiring less time and energy [226]. 

Plant tissues, cellular membranes, and organelles are disrupted, allowing the solvent to enter the cell and dissolve the bioactive compounds [211]. The mass transfer rate is directly proportional to the applied pressure because solubility increases with pressure [229]. As a result, high-pressure treatments improve extraction rates and the availability of bioactive molecules, particularly from difficult-to-release matrices. Pressure has been shown to reduce intracellular pH [230], which aids in the extraction of acylated anthocyanins because they are more stable at low pH [231]. High-pressure treatments also reduce the dielectric constant of water and solvents, which aids in releasing phenolic compounds and the most stable anthocyanins in acylated form, which is less polar [231]. 

#### 5.2.2. Supercritical Fluid Extraction (SFE)

Supercritical fluid extraction (SFE) is another non-conventional extraction method that obeys the principle of the Green Extraction of Natural Products (second principle) [232]. This is due to the use of supercritical fluids such as CO_2_, which can reduce the solvent consumption and amount of waste [233]. SFE operates at temperatures and pressures above the critical points of the solvents used, whereby gas and liquid exist as separate phases. These fluids exhibit the properties of the liquid (density and salvation power) and gas (viscosity, diffusion, and surface tension), facilitating higher extraction yield within a short time [234]. 

Compounds that are soluble in CO_2_, such as oil, fatty acids, carotenoids, and tocopherols, have benefited from the low critical temperature (31 °C) and pressure (7.4 MPa) of CO_2_ [232]; however, due to CO_2_’s low polarity, co-solvents (methanol, ethanol, dichloromethane, acetone, ethylene glycol, water) are sometimes required to extract polar molecules from water-rich FVW [235]. These co-solvents are used to change the polarity of CO_2_, improve its solvating power, and increase the extraction efficiency by reducing interactions between analytes and plant cell matrices [216].

The sample particle size, temperature, pressure, time, co-solvents, solvent-to-solid ratio, and processing before extraction are important SFE operational factors [216]. Pressure and temperature are essential elements in SFE, and adjusting them is critical to achieve optimal yield and economic performance. The performance of SFE is strongly temperature-dependent, as increasing temperature reduces the solvent density, lowering the yield, while increasing the solute vapor pressure increases the yield; however, temperatures that are too high can damage fragile molecules such as carotenoids, altering their structure and bioactivity [236]. Pressure, on the other hand, causes the fluid density to increase as the pressure increases so that the extraction yield improves. In general, high pressure, temperature, and flow rate maximize polyphenol extraction in FVW.

Despite its many benefits, SFE has some drawbacks, including limited solvent diffusibility into the matrix, prolonged extraction times, high pressure requirements, costly infrastructure, inconsistency, and lack of repeatability during continuous processes [234]. Furthermore, even with identical chemicals, SFE process conditions can differ amongst plant matrices. Pre-treatments such as lyophilization, micronization, maceration, and decoction often impact the final extraction yields and compositions; thus, before using SFE for bioactive extraction, it is imperative that the appropriate operating conditions and pre-treatment are thoroughly investigated.

#### 5.2.3. Pulsed Electric Field (PEF)

PEF has been gaining traction for FW recycling and by-products due to its ability to extract valuable ingredients [237]. It is able to decrease energy costs, improve the extraction yield, lessen the degradation of heat-sensitive substances, and purify the extraction process with no environmental impacts [238]. In the PEF process, which occurs at ambient temperatures (20–25 °C), the sample is positioned in the middle of two or more electrodes before being exposed to high-voltage electric field pulses for short processing times with repeated frequency. This results in high electric field strengths (EFS) in batch mode of 100–300 V/cm and in continuous mode of 20–80 kV/cm [239]. 

PEF works by breaking down the structure of plant cell membranes with a high electric field. Due to their dipole nature, the electric charge separates the molecules of plant cell membranes. Because charged molecules repel each other, the pores on the weak sides of the membranes expand, causing permeability [240]. This electroporation or electropermeabilization element allows targeted chemical release from plant matrices [235]. For delicate plant tissues (e.g., pericarp or mesocarp of few fruits), a voltage of 0.1 to 10 kV/cm is sufficient, although for robust materials (e.g., seeds), a voltage of 10 to 20 kV/cm is required [241]. Another benefit of a low EFS (500–1000 V/cm) is the ability of the system to keep the temperature low [242]; thus, PEF reduces heat-labile chemical degradation [243].

The efficiency of PEF-assisted extraction is dependent on the PEF system configuration and extraction parameters. The intensity of the electric fields applied to the processed material is related to the electrode gap, the delivered voltage, the electrode geometry, and their placement in the reactor. Additionally, the extraction yield can be improved by considering the pulse width, number of pulses, treatment time, and total specific energy (kJ/kg). The physicochemical aspects of the treated matrix (size, shape, electric conductivity, cell structure, and membrane characteristics) and nature and cell location of the targeted molecules being extracted can also influence the extraction yield [218].

The EFS affects the physical properties of the targeted molecules, such as their diffusivity, surface tension, viscosity, and solubility [244]. The electric fields must be dispersed consistently across the treatment chamber. There are many waveforms that can be used to deliver electric field energy. In PEF extraction, high-energy exponential square wave pulses are typically used. Due to strong energy transfer in the plant cell matrix, boosting EFS also increases the chemical extraction. The treatment temperature also impacts the PEF extraction process [245], which is commonly conducted at room temperature; however, a high voltage electric field or inefficient delivery pump may increase the overall energy and sample temperature. Higher temperatures may reduce the solvent viscosity, affecting extraction. The treatment time (pulse numbers and width) and solvent selection are equally critical in assessing the PEF performance [128]. An increase in solvent conductivity allows for faster electroporation of the cell membrane. It also aids in mass transfer and increases the extraction rate due to its high solubility in the solvent [246].

Further applications of PEF extraction should be explored; however, the high investment cost is the major hindrance to this technology being widely employed in industry. Nevertheless, its principal benefits outweigh conventional extraction methods; namely, improved extraction yields with minimal thermal degradation while reducing the extraction time, temperature, and solvent usage, subsequently lowering the energy consumption and environmental effects.

#### 5.2.4. Ultrasound-Assisted Extraction (UAE)

Ultrasound-assisted extraction (UAE) is an established extraction method that has been successfully employed to extract polyphenols, carotenoids, volatiles, and polysaccharides from various FVW [117,247,248,249,250,251]. This approach reduces the extraction time (saving energy) and solvent usage while increasing the bioactive component yield from FVW. It is also one of the most common green extraction methods because it is fast and straightforward. UAE utilizes mechanical waves from 20 to 100 kHz [252]. These waves are composed of compression and rarefaction cycles that can travel through any media, displacing and dislodging the treated FVW cell matrix. The cavitation bubbles implode forcefully at the end of the rarefaction cycle, releasing tremendous amounts of energy at temperatures up to 5000 K and pressures up to 50 MPa [253]. The collapsing cavitation bubbles will cause microjets, fragmentation due to rapid interparticle collision, localized erosion, pore creation, shear forces, enhanced absorption, and enhanced swelling index values in the treated plant cells. Reduced particle size, higher surface area, and high mass transfer rates in the border layer of the solid matrix contribute to the solubilization of bioactive components [254]; thus, by improving the mass transfer between the plant cells and solvent, UAE can improve extraction. Despite the considerable energy produced by collapsed cavitation bubbles, the timeframe for these processes is too short to affect the overall system; hence, UAE is the ideal method to extract heat-sensitive compounds [255].

Combining two or more operating factors (frequency, power, duty cycle, temperature, solvent type, and extraction time) creates synergistic effects [256]; thus, determining the extraction kinetics is critical to optimizing extraction times and lowering energy use. In experiments involving sonoporation, capillarity, and detexturization in plant cells, frequency has been shown to have a significant impact on the bioactive chemical yield and characteristics [257]. Low-frequency, high-intensity ultrasound produces strong shear and mechanical forces that are desirable in the extraction process, whereas high-frequency, low-power density ultrasound produces a large number of reactive radicals [247,249,258].

Low frequency is preferred due to large cavitation effects that diminish with ultrasound frequency. Extraction with more than 20 kHz energy affects the physicochemical properties of phytochemicals, causing chemical deterioration and free radical production [259]. Using response surface methods, González-Centeno et al. [248] determined that 40 kHz was most successful in extracting phenolics from grape pomace. The yields were high at both low and high frequencies but low at intermediate frequencies for all examined responses.

The power levels used for UAE bioactives from FVW vary depending on the component to be extracted and the plant matrix chosen for extraction [254]; however, UAE power is inversely proportional to the cavitation bubbles generated within the solvent or solid media. This relationship is significant because as cavitation bubbles collapse, they increase the contact area between the solid and solvent, the shear forces causing turbulence behavior, and ultimately cell wall rupture and solvent penetration. Although increasing the power increases the extraction yield, it should only be increased to a point where the cavitation effects do not diminish. The influence of power on yield also depends on other extraction parameters such as the temperature and solvent extraction time. Achat et al. [260] discovered that the UAE power (60 W) and solvent temperature (olive oil, 16 °C) had significant impacts on oleuropein extraction, with TPC extraction increasing by 53% (414 mg oleuropein eq./100 g). The maximum TPC extraction yield (30.7 mg GAE/g) was achieved by Martínez-Patiño et al. [261] using high amplitude percentages (70%) and extended ultrasonication periods (15 min); however, elevated temperatures (>75 °C) were reached at the end of the experiment, perhaps restricting the extraction yield.

The combined impacts of the sample particle size, solvent-to-solid ratio, pH, temperature, and extraction time on the yields of targeted bioactive chemicals should not be underestimated. More surface area means a higher yield, especially when combined with a higher reaction temperature and the correct solvent-to-solid ratio. The bigger concentration difference improves the solute diffusivity and solubility in the solvent, enhancing extraction [254]. Pectin recovery is high when the pH is low. Insoluble pectin is hydrolyzed into soluble pectin, and the molecular weight of the pectin is reduced, boosting dissolution into the surrounding medium and recovery [262]. The effect of increased sonication time on yield is similar to the effects from increasing the power and temperature. Longer exposure times and higher power input speed up the disintegration of dissolved pectin, producing simpler monosaccharides. According to Wang et al. [262] and Xu et al. [263], increasing the extraction time and decreasing the power intensity (lower energy expenditure) did not improve the pectin extraction yield in grapefruit peels.

It is essential to screen the optimal operating parameters that may help boost the extraction of the targeted chemicals and may further optimize the settings used in prior studies. Furthermore, secondary contamination from the UAE probe should be considered when extracting bioactive chemicals from FVW.

## 6. Conclusions and Future Perspectives

Fruit and vegetable wastes (FVW) from the food bioprocessing industry result in environmental pollution; however, these wastes can be valuable sources of polymer materials. This review focuses on recent advances in biocomposites, active packaging, and by-products and the innovative technologies used for bioactive compound extraction. The mechanical, thermal, antibacterial, and physicochemical properties of FVW-based biocomposites have recently shown improvement. Common matrices used for biocomposites are PLA and PP. Additionally, pectin is an extracted compound used in the polymeric matrix, and the films produced are both active and biodegradable. Additionally, blueberry, sweet potato, and black chokeberry dyes and pigments are added to the films as pH indicators to trace and monitor food freshness throughout storage. To extract bioactive compounds from FVW, various techniques are used, including thermal extraction. Traditional methods (conventional and Soxhlet heating extractions) and new emerging methods (pressurized liquid extraction, subcritical water extraction, and microwave-assisted extraction) have been reviewed in this paper. As a result of the reduced use of organic solvents in the extraction procedures, these well-developed and established methods have been recognized as green technology methods. 

While the use of FVW has the potential to improve polymer properties, it is critical to maintain the low-cost benefits of using FVW while also maintaining the mechanical and thermal properties. Surface treatments with biopolymers or fibers improve these properties but are more expensive. The high temperatures involved in such processes may reduce the quality of the resulting bioactive compounds. The value of FVW for use in biocomposites is expected to grow for both industry and research applications due to global waste concerns. Additionally, the vast majority of FVW-based research has been conducted at the bench scale. The next level of valorization of FVW should be scaling up this process to the industrial level. Overall, the growth of FVW-based polymer materials has been rapid, and their applications in active packaging, biocomposites, by-products, and recent technologies for the extraction of bioactive compounds mean they appear to have a promising future in the coming years.

## Figures and Tables

**Table 1:** Utilization of fruit and vegetable wastes in food packaging systems.

Film Type	Polymers	Fruit/Vegetable Waste	Applications	Findings	Ref.
Active film	Hydroxypropyl high-amylose starch	Pomegranate peel (PGP)	-	PGP inhibited the growth of *S. aureus* and *Salmonella* bacteria.	[34]
Active film	Cassava starch	Blueberry pomace (BP)	-	BP improved the barrier properties and released active compounds into acidic medium.	[39]
Active film	Gelatin capsule waste	Fiber and ethanolic extract from blueberry juice processing waste	Sunflower oil	Improved light barrier properties; reduced the lipid oxidation of sunflower oil and stability in antioxidant activity.	[40]
Active film	Tapioca starch thymol	Jackfruit skin and straw as filler	Cherry tomato	Jackfruit skin and straw resulted in higher tensile strength with lower water solubility and water vapor permeability.	[41]
Active packaging	Poly(butylene adipate-co-terephthalate) (PBAT) Cinnamon essential oil	Cellulose nanofibers (CNF)	Strawberry	High thermal stability, decreased water vapor permeability.	[37]
Active packaging	Chitosan	Apricot kernel essential oil (AKEO)	Bread slices	AKEO improved water resistance, water vapor barrier, and mechanical properties. Inhibited *E. coli*, *B. subtilis* and fungal growth.	[42]
Biodegradable film	Tapioca starch	Banana pseudostems (BP)	-	BP reduced the mechanical and optical properties, improved the barrier properties.	[43]
Active film	Mango kernel fat (MKF), phenolic extract from mango kernel (MKPE), mango kernel starch (MKS),	Mango kernel	-	Films exhibited antioxidant activity, UV-absorbing capacity, and good barrier properties.	[44]
Biodegradable film	Pectin, sodium carboxymethyl cellulose (CMC− Na) Thyme essential oil	Okara soluble dietary fiber	-	Higher mechanical, barrier, optical, and antioxidant properties. Antimicrobial activity against *E. coli* and *S. aureus* was not significant.	[45]
Active edible film	Basil seed gum *Zataria multiflora* essential oil	Basil seed gum (BSG)	-	Efficient antimicrobial activity against *E. coli* and *B. cereus.*	[46]

**Table 2:** Utilization of fruit and vegetable wastes as pH indicator in packaging films.

Polymers	Fruit/Vegetable Waste	Applications	Findings	Ref.
Cassava starch	Blueberry residue (BR) powder, two particle sizes	Distilled water, sucrose, sodium chloride, soybean protein, milk protein, whole milk powder, orange juice, corn oil, chicken meat	BR films displayed changes in color responding to the pH of tested samples.	[50]
Corn starch	Blueberry juice processing waste	-	The blueberry residue film showed visual color differences in different pH ranges.	[51]
Sweet potato starch	Sweet potato peel	Chicken flesh	SPP reduced the mechanical and barrier properties, did not influence the color response of the films.	[52]

**Table 3:** Residues derived from fruit and vegetable waste.

Residue	Fruit/Vegetable	Matrix	Properties	Ref.
Cellulose nanofibrils (CCNF)	Carrot pomace	PLA	Mechanical, hydrophilic, thermal, and antibacterial	[36]
Nanofiber	Durian skin	PLA	Tensile strength	[68]
Fiber	Banana peel	PP	Sound insulation	[69]
Fiber	Durian skin	PLA	Production energy	[70]
Fiber	Durian skin	PLA	Tensile strength, modulus of elasticity, and enzymatic degradation	[71]
Fiber	Sea mango peel	PP	Flexural strength, flexural modulus, and thermal	[72]
Pomace extract	Chokeberry, blackcurrant, apple, and raspberry	Rapeseed meal, microcrystalline cellulose	Flexural strength and water contact angle	[73]
Extract	Coconut shell	Polyvinyl alcohol (PVA) and corn starch	Antioxidant activity and thermal	[74]
Extract	Date fruit	Gelatin	Moisture content and water solubility	[75]
Powder	Cocopith	Wood powder, tapioca	Thermal conductivity	[76]
Powder	Date and tamarind seed	PLA	Tensile, flexural, and impact strength	[77]
Powder	Grape and acerola	Cassava starch	Antioxidant, physicochemical, and mechanical	[78]
Powder and fiber	Jackfruit skin	PLA	Tensile strength and tensile modulus	[79]
Powder	Sour cherry shell	PE	Elastic modulus, tensile strength, moisture absorption, and water vapor transmission rate	[80]
Husks	Chestnut	Starch	Elastic modulus and tensile strength	[81]

**Table 4:** Progress studies of FVW using conventional heating extraction.

Fruit and Vegetables Waste	Bioactive Extraction	Process Condition	Agent	Optimum Yield (%)	Ref.
Red and white dragon fruit peel, passion fruit peel	Pectin	43—107 min, 60–80 °C (Dragon Fruit), 60–120 °C (Passion Fruit)	Citric acid	15.12% (red dragon fruit), 14.11% (white dragon fruit), 13.18% (passion fruit)	[130]
Grape (white and red) skin	TPC	60–90 min, 40–70 °C	Ethanol	1.74–2.12 gGAE/L	[131]
Black carrot pomace	Pectin	90 min, 110 °C	Acidic solution	0.22 kg pectin/kg pomace	[132]
Wheat bran	Phenolics	3, 6, and 24 h, 50–90 °C	99% glycerol, citric acid, Folin–Ciacalteu	4.57–16.11 mgFAE/gdm	[133]
Grapefruit peel	Pectin	90 min, 80 °C	HCl	MW 385.55 kDa	[135]
Lime peel	Pectin	60 min, 95 °C	HCl or citric acid	16.12–23.52%	[136]

**Table 5:** Progress studies of FVW in the application of Soxhlet extraction.

Fruit and Vegetables Waste	Bioactive Extraction	Process Condition	Agent	Optimum Yield (%)	Ref.
Olive Oil (Alperujo) solid waste	TPC	4 h, 70 °C	n-Hexane	0.75–3.76 g/kg raw alperujo	[139]
Indian Soapberry seed	Oil	6 h, 80 °C	n-Hexane	40.63%	[140]
Mango peel, Soursop peel, Grape peel, Grape seed	AOA, TPC, TFC	8 h, 40 °C	60% Ethanol; 1:25 solid/liquid ratio	52.28% (Mango peel), 50.63% (Soursop peel), 64.65% (Grape peel), 18.45% (Grape seed)	[141]
Olive skin/peel	Triterpene acids	unmilled/milled, 30–90 min, 65–70 °C	Ethyl Acetate/Methanol (1:40, 1:20, 1:10 sample/solvent ratio)	unmilled, ratio 1:40 g/mL: 22.24%	[142]
Bitter Gourd peel	AOA, TPC, TFC	6 h, 40–50 °C	Methanol	26.48%	[145]

**Table 6:** Progress studies of FVW in the application of microwave-assisted extraction.

Fruit and Vegetables Waste	Bioactive Extraction	Process Condition	Agent	Optimum Yield (%)	Ref.
Red and White Dragon fruit peels, Passion Fruit peel	Pectin	10–12 min, 75 °C, 153–218 W	Methanol (pH: 2.9–3.0)	17.01 ± 0.32% (Red Dragon Fruit), 13.22 ± 1.42% (White Dragon Fruit), 18.73 ± 0.06 (Passion Fruit)	[130]
Black Carrot pomace	Phenolic, Antioxidants, Anthocyanins	5 min, 110 °C, 20% output power of 900 W	Hot Acidic Water (pH: 2.5)	phenolic content: 1692 ± 79.4 mg GAE/l (0.17 kg pectin/kg pomace); antioxidant: 60 ± 9.6 MTE/mL; anthocyanins: 456.8 ± 38.2 mg/L	[132]
Longan seeds	Pectin	3.5 min, 700 W	50% Ethanol	64.95 + 20.56 mgGAE/gdw	[149]
Lemon, Mandarin and Kiwi peels	Pectin	1–3 min, 60–75 °C, 360–600 W	HCl, Nitric Acid (HCl)	17.97% (kiwi peels), 7.47% (mandarin peels), 7.31% (lemon peels)	[154]
Apple pomace; Orange peel; Mango peel; Carrot pulp	Pectin	10–180 min, 90 °C, 50–200 W	Water	Orange peel, 60 min, 200 W: 12.9 ± 1.0%; Mango peel, 120 min, 200 W: 14.7 ± 0.6%; Apple pomace, 120 min, 200 W: 14.7 ± 0.1%; Carrot pulp, 60 min, 200 W: 6.3 ± 0.7%	[158]
Black Carrot pomace	Phenolic, Flavonoid, Anthocyanins, AOA	9.8 min, 348.07 W	20% Ethanol	polyphenolic content: 264.9 ± 10.025 mg GAE/100 mL; flavonoid: 1662.22 ± 47.3 mgQE/L; AOA: 13.14 ± 1.05 MTE/mL; anthocyanins: 753.40 ± 31.6 mg/L; color density; 68.63 ± 5.40	[161]
Jocote (*Spondias purpurea* L.) pomace	Pectin, Flavonoid	20 min, 68 °C, 100 W	80% Ethanol	phenol: 0.897 g GAE/g (3.42%), flavonoid: 1.271 g QE/g,	[163]
Mango kernel	Crude butter	3.5 min, 160 W	Water	48.85%	[167]
Lemon peel	Essential oil, Pigment	50 min, 20 °C/min, 500 W	80% Methanol	Essential oil: 2 wt.%, Pigment: 6 wt.%	[166]
Watermelon rind	Pectin	12 min, 279.3 W	Acetic Acid	3.93–5.77% (DE: 56.86–85.76%)	[170]

**Table 7:** Progress studies of FVW involving pressurized liquid extraction.

Fruit and Vegetables Waste	Bioactive Extraction	Process Condition	Agent	Optimum Yield (%)	Ref.
Grape pomace: skin, seed	Polyphenols, Antioxidants	5 min with 250 s nitrogen purge, 100–160 °C, ~10 atm	20–60% Ethanol	Polyphenols content, 160 °C, 60% ethanol, Skin:1.98 ± 0.06 mgGAE/gdw; Seeds: 12.54 8 ± 0.02 mgGAE/gdw; Antioxidant by DPPH, 100 °C, 20% Ethanol, skin: 121.91 8 ± 0.08 mg/mL, Seeds: 39.63 8 ± 0.01 mg/mL; Antioxidant by ORAC, 160 °C, 60% Ethanol, Skin: 36.33 8 ± 0.06 MTE/gdw, Seeds: 137.65 ± 0.11 MTE/gdw	[172]
Pomegranate peel	TPC, Punicalagin content, antimicrobial activity	200 °C	77% Ethanol	Polyphenols content: 164.3 ± 10.7 mgGAE/gdw; Punicalagin content: 17 ± 3.6 mg/gdw	[174]
Olive pomace	TPC, AOA, TFC	65–18 °C, supercritical carbon dioxide (scCO_2_)	8–92% Ethanol	TPC, 160.7 °C, 75% Ethanol: 280.37 mgGAE/gDE; AOA, 125 °C, 50% Ethanol: 6.88 MTE/gDE; TFC, 89.3 °C, 25% Ethanol: 15.82 mgRE/gDE	[175]
Mulberry pulp	TPC, Anthocyanins	10 min, 75.5 °C, 200 atm, purge time 90 s	47.2% Methanol (pH3.01)	TPC: 2186.09 ug/g, AOA: 164.53 ug/g	[176]
Beetroot waste: residues, leaves and stems	TPC, AOA	40 °C, 7.5–12.5 MPa, 3 mL/min	70–100% Ethanol	Leaves- TPC, 40 °C, 10 MPa, 100% Ethanol: 252 ± 2 mgGAE/g; AOA by ABTS, 40 °C, 12.5 MPa, 100% Ethanol: 823 ± 48 MTE/g; Stems- TPC, 40 °C, 10 MPa, 100% Ethanol: 14 ± 2 mgGAE/g; AOA by DPPH, 40 °C, 10 MPa, 100% Ethanol: 515 ± 89 G/mL (Leaves > Stems)	[177]

**Table 8:** Progress studies of FVW involving sub-critical water extraction.

Fruit and Vegetables Waste	Bioactive Extraction	Process Condition	Agent	Optimum Yield (%)	Ref.
Grape pomace	Phenolic compounds	50–190 °C	Water	29 g/100 g extracts	[190]
Kiwifruit peel	TPC, TFC, AOA	5–30 min (20 min) 120–160 °C (160 °C)	Aqueous mixture	TPC: 51.24 mg GAE/gdw, TFC: 22.49 mgCE/gde AOA by ABTS: 269.4 mM TE/gdw	[191]
Citrus (*C. unshiu*) peel	TFC	145–175 °C 15 min	Water	TFC: 59,490 g/gdb	[192]
Tamarind seed	Xyloglucan component, TPC, AOA	100–200 °C (175 °C) 5.03–13.55 min	Distilled Water	Xyloglucan component: 62.28%, TPC: 14.65–42.00 gGAE/g, AOA: 1.93–3.20 MTE/g	[193]
Dates seed	TPC, AOA, TFC, Dietary fiber	120–180 °C (144 °C) 10–30 min (18.4 min)	Aqueous mixture	TPC: 9.97 mgGAE/g, TFC: 3.52 mgQE/g, AOA 1.67 mgTE/g, Dietary fibers: 29 g/mg	[194]

**Table 9:** Comparison of innovative extraction methods.

Extraction Method	Working Principles	Parameters	Advantages	Disadvantages
High Pressure Processing (HPP)	Works by increasing pressure up to 600 MPa. Isotactic pressure cause rupture to the cell membrane and increase mass transfer from the inside cell to the outside environment and vice versa [211]. Mild temperature improves further the mass transfer.	Pressure Temperature (mild) Solid/liquid ratio Processing time	Green technology. Good with thermo-sensitive bioactive components	Reduces pressure-sensitive bioactive components [212]. Need to be hurdled with other technologies to improve the extraction process.
Ultrasound-assisted extraction (UAE)	UAE produces acoustic waves in the solvent that lead to cavitation bubbles. The developed cavitation bubbles burst at the surface of the plant sample matrix, disrupt the plant cell wall, and help in the release of the phenolic bioactive compound into the solvent [213]. Suitable for phenolic compounds, lipids, chlorophyll, carotenoids [214]	Concentrations of solvent Solvent-to-sample ratio Extraction time Temperature Frequency Power Particle size Liquid height Duty cycle	Analytically simpler (reduce unit operations), more efficient, lower extraction temperature, and faster processing time [215]. Reduction in energy and power usage. Higher yield and less chemical solvent usage [214]. Conducted under room temperature or heat and at atmospheric pressure. Water, aqueous and non-aqueous solvents. Quick return of investment	Deterioration of phenolic compounds due to the generation of hydroxyl radicals with the formation of cavitation bubbles. Lack of uniformity in UAE energy distribution and potential change in the constitutive molecules [216]. Proper optimization in US frequency, the nominal power of the device, propagation of cycles, input power, system geometry is required for maximum yield [214].
Supercritical fluid extraction (SFE)	A process based on the use of solvents above or near their critical temperature and pressure to recover extracts from solid matrices. Uses supercritical fluids (CO_2_ and H_2_O) for the extraction of phenols from the plant matrices. Suitable for volatile compounds [217].	Temperature Pressure Time Solvent-to-sample ratio Sample particle size Processing before extraction	Faster, selective, and improved recovery of phenolics without using toxic organic solvents [215]. Conducted under room temperature and at high pressure. Automated system, no filtration required, recycle and reuse of the supercritical fluid, polarity of CO_2_ can be retuned and extraction of thermolabile compounds possible at low temperature.	Increased time due to solutes, lower diffusion rate from the solid matrix into the supercritical fluid. Initial cost of the equipment is high. Greatly impacted by the property of the fluid used [215]. Elevated pressure required, risk of volatile compounds losses, many parameters to optimize.
Pulsed electric field (PEF)	Causes electroporation, which involves the formation of localized pores in the cell membranes, increasing the extraction yield [218]. Best for phytosterols and various polyphenols [219].	Electric field strength Treatment time Pulse shape Pulse width Pulse frequency Pulse polarity Temperature Treatment flow mode	Shorter extraction time (μs), consumes less energy, environmentally sustainable [215]. Conducted under room temperature or heat and at atmospheric pressure. Water, aqueous and non-aqueous solvents. Can be applied in continuous mode up to 10000 kg/h [19].	Increases in electric field strength and treatment time could lead to increased energy consumption. High equipment cost [19].

## Data Availability

The data presented in this study are available on request from the corresponding author.
